# 4-Methylesculetin ameliorates LPS-induced depression-like behavior through the inhibition of NLRP3 inflammasome

**DOI:** 10.3389/fphar.2023.1120508

**Published:** 2023-02-23

**Authors:** Khushboo Choudhary, Surendra Rajit Prasad, Kiran Bharat Lokhande, Krishna Murti, Sanjiv Singh, Velayutham Ravichandiran, Nitesh Kumar

**Affiliations:** ^1^ Department of Pharmacology and Toxicology, National Institute of Pharmaceutical Education and Research–Hajipur, Hajipur, India; ^2^ Department of Biotechnology, National Institute of Pharmaceutical Education and Research–Hajipur, Hajipur, India; ^3^ Translational Bioinformatics and Computational Genomics Research Lab, Department of Life Sciences, Shiv Nadar University, GBNagar, Uttar Pradesh, India; ^4^ Department of Pharmacy Practice, National Institute of Pharmaceutical Education and Research–Hajipur, Hajipur, India

**Keywords:** 4-methylesculetin, anti-depressant activity, NLRP3 inflammasome, lipopolysaccharide, cytokines

## Abstract

The pathophysiology of depression is heavily dependent on inflammation. Evidence suggests that the etiology of depression is linked with NLRP3 inflammasome-induced inflammation. Therefore, blocking the activated NLRP3 inflammasome may be beneficial for treating depression. Due to the limitations of currently available antidepressants, it is necessary to develop novel, safe, and affordable drugs for the treatment of depression. A natural coumarin derivative named 4-methylesculetin (4-MESC) possesses anti-inflammatory properties. However, the role of 4-MESC as an antidepressant has not been elucidated. Therefore, in this study, we explored the antidepressant-like effects of 4-MESC and its underlying molecular mechanism through the modulation of the NLRP3 inflammasome. The docking and molecular dynamic simulation studies revealed that 4-MESC has a higher affinity for the NLRP3 PYD. Blood–brain barrier permeability was confirmed using the SwissADME pharmacokinetic tool. High doses (50 mg/kg) of 4-MESC significantly reduced the immobility duration in the tail-suspension test (TST) and forced swim test (FST) without changing the overall locomotor activity in the female Swiss albino mice that were subjected to lipopolysaccharide (LPS). LPS-induced pro-inflammatory cytokines such as IL-6 and TNF-α were reduced in serum and brain tissues using 4-MESC. 4-MESC’s neuroprotective effects are mediated by increased brain-derived neurotrophic factor (BDNF) and decreased cortisol levels. 4-MESC markedly reduced LPS-induced elevated levels of ROS and lipid peroxidation (malondialdehyde levels) and enhanced the superoxide dismutase (SOD) activity and glutathione levels, which revealed its anti-oxidant potential against oxidative stress. 4-MESC diminished the expression levels of NF-κBp65, IL-6, NLRP3, caspase-1, gasdermin D, and IL-1β in the hippocampus. These findings demonstrated that 4-MESC exhibited antidepressant-like effects by inhibiting the NLRP3 inflammasome. However, other antidepressant mechanisms might also be involved which require further studies.

## Introduction

Depression is a low-mood situation that happens in everyone’s life and impacts the work efficiency of most affected individuals. It is very common among people of all ages and genders in varying degrees of severity. Depending on the severity of the disease, different people experience different symptoms. The most common symptoms are mood swings ranging from emotional bursts to anger, reduced food intake, sometimes overeating, disturbed sleep, an inability to focus on work, a sense of guilt, hopelessness, and loneliness, which can lead to suicidal ideation if left untreated. Depression has become a common disorder worldwide, affecting an estimated 3.8% of the population, including 5% of adults and 5.7% of adults over the age of 60 (World Health Organization, 2022). Depression affects approximately 280 million people, and the numbers exceeded 300 million in 2015, which was equivalent to 4.3% of the world’s population (World Health Organization, 2022). According to the National Mental Health Survey 2015–16, one in 20 Indians suffers from depression, and approximately 15% require active intervention for this mental health issue (World Health Organization, 2023). At its worst, depression can result in suicide; every year, over 800,000 individuals commit suicide, and it is the second leading cause of death among people between ages 15–29 years (World Health Organization, 2023). According to estimates, approximately 258,000 suicide cases occurred in India in 2012 among people in the age group of 15–49 (World Health Organization, 2023). It is predicted by the WHO, after examining the existing scenario of depression among people, which currently holds the third rank for the burden of disease, that it may progress to rank first by 2030 (Malhi and Mann, 2018). The progression of depression accelerated during COVID-19, when the entire nation was in lockdown, due to the deaths of numerous people (Santomauro et al., 2021). Many factors are associated with the pathophysiology of depression. The most popular biochemical theory is the monoamine hypothesis, which states that a deficiency of monoamines (norepinephrine, serotonin, and dopamine) leads to depression (Millan, 2004). The monoaminergic system covers a large area of the brain, which suggests that this system is involved in many brain functions. In patients with depression, many of these functions are impaired (Dunlop and Nemeroff, 2007). The stresses of life also contribute to depression. Recurrent stress is an important factor promoting the pathogenesis of depression. Depression can, in turn, lead to more stress and dysfunction and worsen the affected person’s life situation (Ben-Azu et al., 2020). In contrast, depression can lead to a higher risk of developing inflammatory disorders, such as diabetes and cardiovascular disorders (Okoh et al., 2020). There is a strong link between aberrant immune functions and depression. It has been noticed for a long time that repeated stress also induces acute changes in the immune system. In the case of stress conditions, the hyperactivation of the corticotropin-releasing factor (CRF) occurs which activates the hypothalamic–pituitary–adrenal (HPA) axis and leads to depression (Gold et al., 2002). Inflammatory responses play a vital role in the pathogenesis of depression. Many studies have found that depressed people have higher levels of pro-inflammatory cytokines (Dantzer et al., 2008). Malondialdehyde (MDA) levels and depression are interlinked. MDA levels are increased in depressed mice compared to normal mice (Pal et al., 2006). Also, gamma-aminobutyric (GABA) levels decreased in the prefrontal and occipital cortex in the depressed patient (Petty et al., 1992). The effect of inflammation and inflammatory cytokines on the monoamines, and the excitatory amino acid glutamate, has received attention because neurotransmission plays an important role in mood regulation. Inflammatory cytokines can reduce synaptic availability of monoamines in various ways, which is thought to be a fundamental factor in the pathogenesis of depression (Miller and Raison, 2016). For instance, in experimental mice, it has been demonstrated that IL-1b and TNF-α activation of p38 mitogen-activated protein kinase (MAPK) increases the expression and functionality of the serotonin reuptake pumps, resulting in the decreased synaptic availability of serotonin and depressive-like behavior (Zhu et al., 2010). Inflammatory cytokines have also been reported to reduce the availability of tetrahydrobiopterin (BH4), an essential enzyme co-factor in the production of all monoamines that is extremely vulnerable to oxidative stress, by producing reactive oxygen and nitrogen species (Neurauter et al., 2008) The treatable drugs mostly have mechanisms for regulating the monoamine levels, which are not adequate for an effective cure, and the conventional drug is the only available treatment for depression, which has certain limitations such as nausea, sexual dysfunction, insomnia, anxiety, dry mouth, and weight gain (Hu et al., 2004). Using the Antidepressant Side-Effect Checklist (ASEC), the reported side effects of antidepressant drugs were categorized as mild, moderate, and severe. A total of 74.13% patients reported antidepressant side effects, with anxiety (17.05%) and sleepiness (17.05%) being the most common ones. More than half of the patients, 52.29%, exhibited low levels of adherence (Marasine et al., 2020). Therefore, the development of safe and effective antidepressants of natural origin is necessary as an alternative therapy. It has been observed in many studies that there is a link between inflammation and depression (Tong et al., 2020). TNF-α, IL-1b, and IL-6, pro-inflammatory cytokines, were more prevalent than usual in clinical samples from depressed patients (Amitai et al., 2016). Additionally, elevated inflammatory mediators may act as indicators that predict antidepressant resistance. Therefore, anti-inflammatory drugs can actually be beneficial for MDD patients who are treatment-resistant and have higher levels of inflammatory mediators (Tong et al., 2020). Recently, the inflammasome has become an unanticipated sensor for metabolic stress and risk (Alcocer-Gómez and Cordero, 2014). The NLRP3 inflammasome is a composite protein mostly composed of three components, namely, the NOD-like receptor NLRP3, central connector ASC, and caspase-1. The effectiveness of this integrated system is to provide protection against the invading microbes *via* the activation of inflaming mediators such as interleukins (ILs: IL-1b and IL-18, further leading to pyroptosis) (Amitai et al., 2016). The pyroptosis-related protein gasdermin D is cleaved, the pro-inflammatory cytokine IL-1b is produced, and microglia are activated as a result of NLRP3 inflammasome activation (Fu et al., 2013). The NLRP3 inflammasome has been linked to the pathophysiology of depression and may offer a fresh approach to treating the condition. Patients with depression had elevated levels of NLRP3, caspase-1, and IL-1b in their blood samples (Alcocer-Gómez et al., 2014). It has been observed that rodent depression models activate the NLRP3 inflammasome (Alcocer-Gómez et al., 2017). Suppression of the NLRP3 inflammasome improved the depressive-like behavior in mice and decreased IL-1b expression (Xu et al., 2016). Interleukin (IL)-1b, a pro-inflammatory cytokine, was the first cytokine to be identified as enhancing HPA axis activity during an immunological response (Bernton et al., 1987). There are many studies that show depression is associated with increased brain or peripheral IL-1b production, as well as HPA axis hyperactivity (Maes et al., 1993). The report suggested that the NLRP3 inflammasome may be a key mediator of stress-induced depression and a potential novel target for depression as a result of the NLRP3 inflammasome’s role in lipopolysaccharide (LPS)-induced depression (Iwata et al., 2013).

Natural coumarin anti-oxidant 4-methylesculetin (4-MESC) has anti-inflammatory effects on intestinal inflammation, whose pathogenesis and oxidative stress are both major contributors to it (Tanimoto et al., 2020). 4-methylesculetin is naturally derived from the peels of *Aesculus hippocastanum* L. It is 6,7-dihydroxy-4-methylcoumarin and can be synthetically derived from other coumarin derivatives. Trinitrobenzenesulfonic acid (TNBS) caused intestinal inflammation, which 4-methylesculetin (4-MESC) prevented by suppressing myeloperoxidase (MPO) activity and preventing glutathione (GSH) depletion (Witaicenis et al., 2010; Witaicenis et al., 2012). In fact, the intestinal anti-inflammatory effects of 4-MESC were also associated with a decrease in malondialdehyde levels *in vivo* and the suppression of a number of pro-inflammatory cytokines *in vitro*, including IL-1, IL-8, IL-2, and IFN-γ (Witaicenis et al., 2012). When compared to esculetin, another coumarin derivative, 4-MESC’s anti-oxidant, and anti-inflammatory properties were dependent on the presence of a methyl group at the C-4 position (Zhao and Zhao, 2020). Although its anti-oxidant and anti-inflammatory activity are reported, until date, there is no report of 4-MESC as an antidepressant agent. The present study was designed to assess the anti-depressant effect of 4-MESC and its underlying mechanism through the inhibition of NLRP3 inflammasome activation induced by LPS administration.

## Materials and methods

### Reagents

LPS (from *Escherichia Coli* 0111: B4) for the *in vivo* study was purchased from Sigma. Mouse Il-6 ELISA Kit, Glutathione Assay Kit, Mouse Tumor Necrosis Factor-alpha ELISA Kit, and SOD Activity Assay Kit were purchased from Sigma. Mice cortisol and BDNF ELISA Kits were purchased from MyBioSource. A BCA protein assay kit was purchased from Merck, and 4-methylesculetin (4-MESC), from TCI Chemicals.

### *In silico* study

#### Docking and molecular dynamic simulation study

The docking and MD simulation were used to check the interaction between receptor and ligand, as described by Lokhande et al. (2021). The Protein Data Bank was used to find the X-ray crystal structure of the NLRP3 pyrin domains in humans (PDB ID: 3QF2). The SDF file of the structure was retrieved from PubChem. Its energies were reduced using the OPLS-2005 force field and Maestro Schrodinger software until an energetically stable conformation was attained. For a molecular-docking investigation involving the NLRP3 pyrin domain, these energetically stable conformations were used. Small compounds are docked into the protein-binding site using molecular-docking techniques. FlexX software was used for docking studies on natural chemicals to determine how these medications interact with the NLRP3 pyrin domain. Receptor atoms from the PDB database, for example, the NLRP3 pyrin domain (PDB ID: 3QF2), are treated as stiff during docking computations. The binding cavity in the receptor is categorized using the receptor preparation wizard option available in FlexX software. Then, all substances are docked to the NLRP3 pyrin domain’s binding site to forecast the binding poses of the retrieved medications. This was performed to assess the docking study’s results in the binding pocket of the pyrin domain, which were revealed under conformational dynamics, and the binding potency of crystal ligands and 4-MESC. 4-MESC was the primary natural chemical that we simulated by using full-scale molecular dynamics for 100 nanoseconds using Desmond. The extended simple point charge (SPC), a three-point water model with periodic boundary conditions, is used to immerse the system for complexes in a cubic box filled with water and having water molecules spaced at a distance of one. By adding the proper counter ion randomly to the solvated complex system, the entire charge of the solvent system is neutralized. The steepest descent method is used to minimize energy, which is an essential stage in MD. When applying periodic boundary conditions to a finite system, edge effects are minimized using a cubic box type with a box size of 0.9. Translated copies, which cover the space-filling box, are filled with the atoms of the system that will be replicated. In this work, the OPLS-2005 force field, an enhanced force field suitable for MD simulation of the receptor, is used to characterize the system’s potential energy. The Martyna–Tobias–Klein barostat method and the Nose–Hoover chain thermostat method are used in molecular dynamic investigations, which take into account a number of parameters as inputs. The constraints are established as all-bonds. The trajectories of stable conformation are recorded and evaluated to investigate relationship stability after the system has reached equilibrium. The initial conformations and the C-alpha backbone conformational change of the NLRP3 pyrin domain crystal structure have been compared.

#### Pharmacokinetic measurement

The pharmacokinetic (ADME) characteristics of the substances have been evaluated using the SwissADME online approach. The five principles of Lipinski (molecular weight not exceeding 500 Da, not more than five H-bond donors, not more than 10 H-bond acceptors, molar refractivity between 40 and 130, and LogP value not exceed 5 have been taken into account for the assessment of favorable drug-like characteristics of any substance.

### *In vitro* study

#### DPPH assay

DPPH solution (1 mg/ml) was prepared in methanol. In a 96-well plate, 50 µl of the DPPH was placed with or without the drug. 4-MESC was added in the concentration range of 25, 50, 100, 200, and 500 µM. The standard anti-oxidant ascorbic acid was taken as the positive control (200 µM). After that, it was incubated at room temperature under dark conditions for 1 h. After incubation, the absorbance was read at 517 nm using a microplate reader (BioTek/Synergy H1). The decrease in absorbance was considered DPPH scavenging activity (More & Makola, 2020).

#### MTT for cytotoxicity assay

To assess the toxicity of 4-MESC, the MTT assay was performed on the RAW 264.7 cell line. RAW cell lines (1 × 10^6^ cells/ml, per well) were seeded in the 96-well plate and kept for 24 h at 37°C in a CO_2_ incubator. After that, drugs were added in the concentration of 0.5, 1, 1.5, 2, 2.5, and 3 mM and incubated for further 24 h under the same condition. After appropriate incubation, the MTT (5 mg/ml, 10 µl) was added to each well and incubated for 4 h at 37°C. DMSO (100 ul) was added to the well to stop the reaction, and after 30 min, with the help of a microplate reader (BioTek/Synergy H1), absorbance was recorded at 570 nm (Sangaran et al., 2021).

#### Determination of reactive oxygen species

The ROS from the C6 cell line were estimated using the H_2_DCFDA dye. The C6 cell line (1 × 10^6^ cells/ml, per well) was seeded in a 96-well plate and incubated for 6 h in a CO_2_ incubator with or without the drug. After that, the H_2_DCFDA dye (20 µM) was added to each well and kept for 30 min at room temperature. The fluorescence was measured at Ex-495/Emi-530 nm using a microplate reader (BioTek/Synergy H1) (Baek et al., 2020).

### *In vivo* study

#### Animals and housing

All animals were shipped from Central Drug Research Institute (CDRI), Lucknow. Adult female Swiss albino mice weighing 25–30 g (6–8 weeks old) were acclimatized for weeks before starting the experiment. Mice were kept under a controlled environment with conventional laboratory settings, including 25°C ± 2°C ambient temperature and an equal shift of light and dark exposure. Mice were fed a regular pellet diet and were given tap water. The guidelines for performing experimental work on animals were approved by the Institutional Animal Ethical Committee (IAEC Approval No. NIPER-H/IAEC/03/21).

#### Experimental outline

A total of 30 female Swiss albino mice were chosen and segregated into five different groups (n = 6). Animals were grouped as follows: normal control (group 1), LPS control (group 2), pretreatment group in low-dose treatment (4-MESC + LPS; group 3), pretreatment group in high-dose treatment (4-MESC + LPS; group 4), and standard group (fluoxetine + LPS; group 5). The experimental plan was carried out for 28 days. Mice of group 1 received normal water. The pretreatment of 4-MESC with low doses (25 mg/kg) and high doses (50 mg/kg) were administered orally to the third and fourth groups, respectively, for 28 days. Fluoxetine (20 mg/kg p.o.) was administered to mice for 28 days as a standard drug. On the 28th day, all mice except those in the control group received a single injection of lipopolysaccharide (0.83 mg/kg i.p.). The day after LPS administration, all mice in each group were evaluated behaviorally using an open-field test (OFT), an immobility test using a tail-suspension test (TST), and a forced swim test (FST). All animals were anesthetized, and retro-orbital blood was withdrawn. Then, the animal was decapitated. The serum and brains of mice were stored at −80°C for biochemical estimations.

#### Behavioral experiments

Various behavioral parameters were assessed in the open-field test, tail suspension test, and forced swim test after 24 h of LPS administration.

#### Open-field test

The formal experiments need to be carried out before the open-field test. This test is designed to determine whether the reported side effects of an antidepressant drug are the result of stimulating general motor activity in rodents exposed to unfamiliar surroundings. Mice were individually subjected to an IR actophotometer chamber, which was cleaned and free of bedding and litter. The chamber was divided into nine virtual quadrants, and mice were dropped at predetermined locations for free movement. The locomotor activity of mice was observed by counting the crossings of virtual quadrants and stretching their forelimbs to climb during a 5-min period (Zhao et al., 2019).

#### Forced swim test

This test is widely used for investigating the behavior of the rodent model of depression and to examine the antidepressant-like effect of novel drugs in rodent models. When mice were forcefully subjected to water for an extended period, they exhibited recognizable immobile behavior. The antidepressant medications reduced the immobility behavior while increasing escape actions such as climbing and swimming. Mice were placed in a transparent cylinder and allowed to swim for 6 min in an experimental test. Video tracking of mice was performed using the ANY-maze software, and the immobility phase was considered for only the last session of 4 mins. Depressed mice showed very little movement and did not try hard to escape that zone. Also, they float motionless by simply keeping their head and noses above the water. Immobility behavior was calculated as the duration of time in which the animal did not show the basic movement to escape. Following the test, the mice were removed from the water and placed in another empty cage, where they were wiped down and dried with a hair dryer. After complete drying, the animal was left in its home cage. The water was changed between testing sessions (Ko et al., 2014).

#### Tail-suspension test

This test functionally resembles the FST. Before induction with stress, acute antidepressant therapy shortens the TST’s immobility period, which is thought to have strong predictive validity. Individual mice were hung by the tail with a fixing tape at the edge of the hook of the burette stand for 6 min. Out of the 6 mins of experimental duration, the immobility phase was recorded for the last 4 min only. The immobility phase of the mice was observed only when they hung passively and stayed motionless (Gill et al., 2018; Redza-Dutordoir and Averill-Bates, 2016).

#### Estimation of superoxide dismutase activity

The activity of the superoxide dismutase (SOD) enzyme was performed according to the method described in the SOD activity assay kit (Sigma). Around 50 mg of tissue was washed with PBS and homogenized in 500 µl of RIPA buffer, protease, and phosphatase inhibitors. After that, tissue lysates were centrifuged at 14,000 g for 10 min at 4°C. The tissue supernatant was pipetted out in the fresh tube and proceeded further as instructed by the kit protocol. After appropriate incubation, the absorbance was noted at 450 nm using a microplate reader (BioTek/Synergy H1).

#### Measurement of the glutathione level

The level of glutathione was estimated as described in the glutathione assay kit (Sigma). The tissue samples were washed with PBS, and 100 mg of tissue was treated with three volumes of 5% sulfosalicyclic acid (SSA) solution (300 µl) and vortex. Then, we added another seven volumes of the 5% sulfosalicyclic acid (SSA) solution and homogenized it. After 10 min at 2°C–8°C, the sample was centrifuged at 10,000 *g* for 10 min. The supernatant was collected and processed further as instructed. Appropriate dilution was performed to stay within the detection range. The absorbance was read at 412 nm by a microplate reader (BioTek/Synergy H1).

#### MDA level estimation

The oxidative injury of lipids by the interaction of free radicals with phospholipids inside a cell was determined using an MDA assay kit (Sigma). Thiobarbituric acid (TBA) and MDA react to produce colorimetric or fluorometric by-products that are equivalent to the amount of MDA present, which is used to measure lipid peroxidation. Tissue samples (10 mg) are washed in PBS before being homogenized with a 100x mixture of MDA lysis buffer (300 μl) and BHT (3 μl). The samples were processed at 13,000 g for 10 min, and 200 µl of supernatant from each sample was pipetted out for further processing. The relative fluorescence unit was measured at EX/EM-532 nm/553 nm by a microplate reader (BioTek/Synergy H1).

#### Estimation of pro-inflammatory cytokines, BDNF, and cortisol level by ELISA

According to the instructions provided by the manufacturer, an ELISA kit was used to quantify the levels of pro-inflammatory cytokines, cortisol, and BDNF in both samples (serum and brain tissues). Samples were prepared according to the instruction, and results were shown as pg/ml or ng/ml as per requirement.

#### NLRP3 inflammasome estimation in gene expression studies using semi-quantitative PCR

In this procedure, 100 mg of whole hippocampal tissue was mixed with 1 ml of TRIzol and allowed for tissue lysis. After homogenization, lysates were allowed to stand for 5 min at room temperature. Then, 200 μl of chloroform was added, and the sample was vigorously shaken for 15 s before being allowed to stand for 15 min. The mixture was centrifuged at 12,000 x g for 15 min at 4°C, separating it into three distinct phases. The top aqueous, colorless phase containing RNA was collected in another sterile tube that had been treated with diethyl-pyrocarbonate (DEPC), to which 500 μl of chilled isopropanol was added and set aside for 10 min. Then, it was centrifuged at 4°C (12,000 x g; 10 min), which resulted in a total RNA pellet at the bottom of the tube. The supernatant was discarded, and the pellet was washed by adding 1 ml of 75% ethanol/ml of TRIzol. To obtain total RNA, the sample was vortexed and centrifuged at 4°C (7500 x g; 5 min). At last, the supernatant was removed, and the final RNA pellet was left for air drying. For longer storage, this RNA pellet was resuspended in 30 μl of RNase-free water with gentle mixing with a pipette and stored at −80°C. The yield of RNA in terms of concentration and purity was determined by the absorbance method (260/280) using NanoDrop (Thermo Fisher), and agarose gel was used further for visualizing the two bands of different sizes of an RNA sample for more clarity of the yield. As per the manufacturer’s protocol, cDNA was prepared using the Verso cDNA Synthesis Kit. The sample preparation should be carried out on ice. Except for reverse transcriptase, all of the kit’s reagents were thawed on ice and gently spun after being removed from −20°C. The reaction volume was set to 20 μl, and the reagents were added in the following order: nuclease-free water: 12 μl, 5x iScript selected reaction; Mix: 4 μl, Control RNA (up to 1 μg): 1 μl, and oligo (dT) primer: 2 μl; and lastly added iScript reverse transcriptase: 1 μl. The final volume of the reaction was mixed, and the parameter was set (70 min; 42°C for oligo dT). Then, reverse transcriptase was heat-inactivated at 85°C for 5 min. The cDNA product was stored at −20°C. The semi-quantitative PCR was set for the genes (NLRP3, caspase 1, ASC, gasdermin D, IL-1β, Nf-κBp65, IL-6, GAPDH), which contained 100 μl reaction volume consisting of 1x PCR buffer, a variable concentration of MgCl_2_, cDNA, forward and reverse primers, 200 µM of dNTPs, and volume makeup with water. Gene amplification was carried out for 28 cycles, initializing with 2 min of initial denaturation (95°C), final denaturation (95°C; 30 s), annealing temperature (55°C–60°C), extension temperature (72°C; 30 s), and final extension (72°C; 7 min). The final amplified product of the gene was stored at 4°C, and it was run on 1% agarose gel with the loading control as GAPDH and visualized in ChemiDoc (Bio-Rad).

#### Evaluation of the NLRP3 inflammasome inhibition of 4-MESC through western blot

A total of 100 mg of mice brain tissue was taken from each group and homogenized in 500 μl of RIPA buffer with an additional protease inhibitor and phosphatase inhibitor at 4°C. The homogenized sample was allowed to stand for 20 min and centrifuged to 10,000 g for 15 min at 4°C. The supernatant was withdrawn and collected in other tubes. The BCA protein assay kit was used to estimate the protein concentration in the supernatant. A total of 60 μg of total tissue lysate was taken and mixed with the SDS loading dye, boiled for 5–10 min, and then centrifuged and loaded in a 10% SDS page. The protein on the SDS page was then transferred into a PVDF membrane (0.45 μm) for 1.5 h. The PVDF membrane was blocked with 3% BSA in 1% TBST for 1 h and incubated overnight at 4°C with the following primary antibodies: caspase 1(1:2000, Invitrogen), Nf-κBp65 (1:2000, Sigma), NLRP3 (1:1000, Invitrogen), and *β*-actin (1:5000, Thermo Fisher). The next day, the same PVDF was washed five times with TBST at 5-min intervals and incubated 1 h with the Horse redox peroxidase (HRP)-conjugated secondary antibody goat anti-mouse IgG (1:10,000, Thermo Fisher) or goat anti-rabbit IgG (1:10,000, Thermo Fisher). The blots were then visualized with ECL chemiluminescent substance in ChemiDoc (Bio-Rad).

#### Measurement of the NLRP3 level

The level of NLRP3 was estimated as described in the NLRP3 assay kit (Abcam). A total of 100 mg tissue was homogenized with 500 μl of 1x cell extraction buffer and incubated in ice for 20 min. Then, it was centrifuged at 18,000 g for 20 min at 4°C. The supernatant was collected and proceeded for further estimation. The absorbance was taken at 450 nm using a multimode reader.

### Statistical analysis

Statistical analysis was carried out by one-way analysis of variance (ANOVA) using GraphPad Prism software (version 5.00; GraphPad software Inc., La Jolla, CA, United States). The results were shown as mean ± SD values. Differences between group data were considered statistically significant at *p* < 0.05.

## Results

### Docking and MD simulation study reveals high affinity to NLRP3 PYD

**FIGURE 1:**
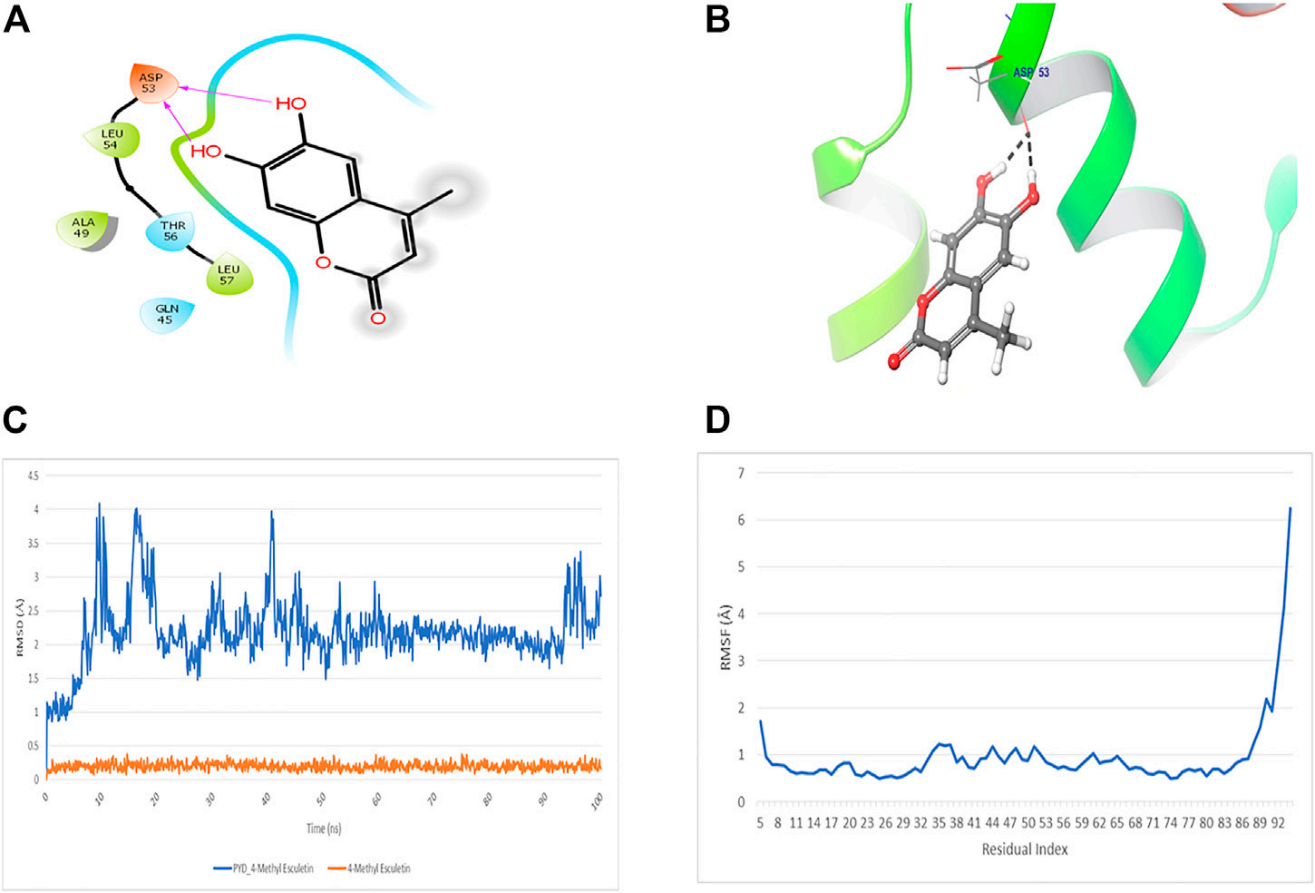
Docking and MD simulation study of 4-MESC with NLRP3 PYD. **(A)** 2D image of binding position of 4-MESC within the binding pocket of NLRP3 PYD. **(B)** 3D image. **(C)** Root mean square deviation of the C-α backbone of NLRP3 PYD after binding with 4-MESC. **(D)** Root mean square fluctuation of the C-α backbone of NLRP3 PYD after binding with 4-MESC.

The docking study of 4-MESC and NLRP3 PYD was performed to check the affinity. The docking score was found to be −9.879. The binding mode of 4-MESC in the NLRP3 PYD interacting pocket is shown in Figure 1A (2D) and Figure 1B (3D). 4-MESC formed two hydrogen bonds with NLRP3 PYD. An intermolecular force called the hydrogen bond held two or more molecules together. The amino acid Asp 53 was involved in hydrogen bonding with 4-MESC. A docking score of −10 and below using Glide SP is considered to be a good binding for the ligand with protein. In the present study, the binding score was −9.879, which reflected a good binding of 4-MESC with NLRP3 PYD. Since the NLRP3’s rigid crystal structure was used for molecular-docking simulations, interaction between the target receptor and 4-MESC was examined using molecular modeling, and dynamic behavior of the receptor and ligand was used to test the stability of bound conformation using dynamic simulation following compound chemistry in the pocket of NLRP3 PYD. To investigate the protein–ligand conformational stability, we have simulated the changes in complexes and their systems each up to 100 ns In this investigation, the time period which is a sufficient time for the rearranging of NLRP3 PYD’s Cα atoms in complexes with 4-MESC. Root mean square deviation (RMSD) and root mean square fluctuation (RMSF) were calculated to demonstrate thermodynamic conformational stability during a 100-ns time frame. An additional 1,000 trajectories were gathered and stored during the MD simulation. The NLRP3 PYD’s original crystal structure was overlaid using the SEA panel for the simulation event analysis of Schrodinger’s equation, which produced data in the “. dat” format. The RMSD and RMSF graphs were created using the collected RMSF and RMSD values. The RMSD plot suggested that 4-MESC had a mean RMSD value of 2.19 Å which is an acceptable range of RMSD (Figure 1C), indicating that it is strongly bound to NLRP3 PYD. On the other hand, the RMSF values for Cα atoms present in all the residues were calculated to investigate the binding effectiveness of compounds with NLRP3 PYD, based on data from 100 ns of trajectories. The NLRP3 PYD following the binding of 4-MESC, the average RMSFs are 0.95 Å (Figure 1D) which shows the least amount of variation and the NLRP3 relative secondary conformational stability upon the described chemicals’ binding. Therefore, the MD research revealed that the 4-MESC had greater potential toward NLRP3 PYD. The pharmacokinetic study using the SwissADME online tool showed that this particular compound followed the Lipinski rule of five (molecular weight: 192.17 g/mol, number of H-bond acceptors: 4, number of H-bond donors: 2, Log *p*-value is 1.45, and molar refractivity is 51.50) and claimed its high GI absorption and blood–brain barrier permeability. It is an inhibitor of cytochrome *CYP1A2* enzyme which will increase the plasma concentrations of the drug. The bioavailability score of the 4-MESC was found to be 0.55 which shows good bioavailability. The LogP value, for oral and intestinal absorption of drugs should be ideally between 1.3–1.8; and for 4-MESC, it is 1.52 which shows its drug -likeness.

### Anti-oxidant and cytotoxicity activity of 4-methylesculetin

**FIGURE 2:**
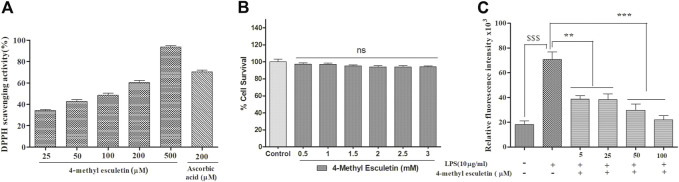
Anti-oxidant activity was evaluated by DPPH scavenging, ROS estimation and cytotoxicity by MTT. **(A)** Anti-oxidant efficacy was evaluated by the DPPH assay with an increasing concentration of 4-MESC and ascorbic acid taken as a positive control. **(B)** Cytotoxicity of 4-MESC was evaluated on the RAW cell line up to 3 mM concentration by an MTT assay. **(C)** ROS were estimated by an H_2_DCFDA dye after the LPS treatment and along with the different concentrations of 4-MESC. Data were represented as mean ± SD and *p < 0.05, ** 0.05 < *p* < 0.01, and *** 0.01 < *p* < 0.001 were considered significant compared to the LPS control.

The anti-oxidant effect of 4-MESC was assessed using a DPPH assay. This assay revealed that the DPPH scavenging activity of 4-MESC increased with increasing concentration. Almost 90% of DPPH was scavenged by 4-MESC at 500 µM concentration (Figure 2A). The standard anti-oxidant compound ascorbic acid was taken as the positive control and showed ∼65% scavenging activity at 200 µM (Figure 2A). The cytotoxicity of 4-MESC was assessed by an MTT assay on RAW 264.7 cell lines. The 4-MESC was treated at concentrations ranging from 0.5 mM to 3 mM. The cytotoxicity assay showed that it was not cytotoxic at even high concentrations up to 3 mM (Figure 2B), revealing that it was minimal or not cytotoxic for normal cells. We estimated the ROS production inside the LPS and LPS with 4-MESC-treated C6 cells. We found that ROS production was increased in cells treated with LPS (*p* < 0.001) compared to control cells, but significantly reduced with an increasing concentration of 4-MESC. Almost 60% of ROS production was decreased in 4-MESC and LPS-treated cells at 100 µM concentration compared to LPS-treated cells (*p* < 0.001) (Figure 2C).

### Effect of 4-methylesculetin on locomotor activity

**FIGURE 3:**
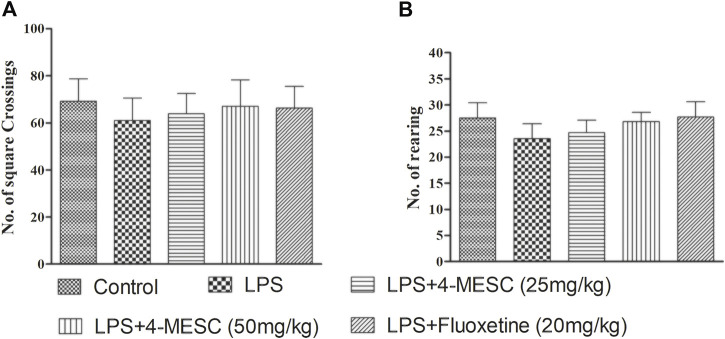
Effect of lipopolysaccharide (LPS) and 4-methylesculetin on autonomous activity were evaluated by an open-field test (OFT). **(A)** No. of crossing. **(B)** No. of rearing. Data are expressed as mean ± SD. The results showed no difference between the different groups.

The LPS and 4-MESC groups (25 mg/kg and 50 mg/kg) did not show any comparable differences in the movement pattern (number of squares crossing and number of rearing) as compared to the normal control group (Figures 3A, B). Moreover, there was little reduction in motion in LPS-treated mice, but the difference was not significant compared to the control. This result showed that the antidepressant effect of 4-MESC was not due to stimulation of the general motor activity.

### Effect of 4-methylesculetin on immobility time in the forced swim test and tail-suspension test

**FIGURE 4:**
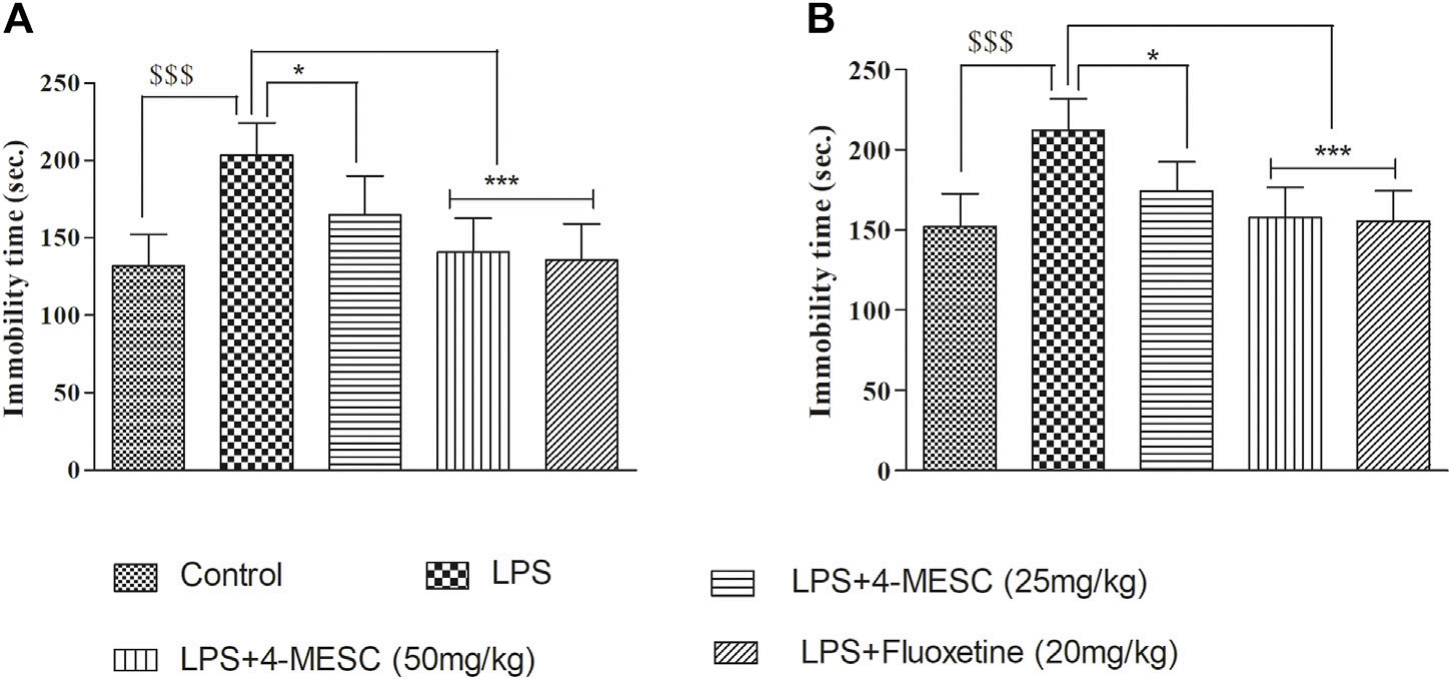
Effect of 4-MESC on immobility time. **(A)** Forced swim test (FST). **(B)** Tail-suspension test (TST). Data were represented as mean ± SD. ^$$$^ *p* < 0.001 compared to the control and *p < 0.05, ** 0.05 < *p* < 0.01, and *** 0.01 < *p* < 0.001 were considered significant compared to LPS.

In the present study, LPS-induced stress which caused depression-like behavior, and it was evaluated using the immobility time in the FST and TST. In the FST, our results showed that LPS-induced animals showed less movement with their paws and a significant (*p* < 0.001) increase in immobility duration compared to that of the normal control group. Similarly, in the TST, the immobility time is increased in the LPS group as compared to the control group (*p* < 0.001). These findings reflected the successful development of the LPS-induced depression model in mice. Pretreatment with 4-MESC reversed the LPS-induced increased immobility duration at both dosages, 50 mg/kg (*p* < 0.001) and 25 mg/kg (*p* < 0.05), significantly in FST and TST studies. Likewise, the standard group (fluoxetine) also showed significant (*p* < 0.001) decrease in the immobility time as compared to the LPS group. Findings imply that 4-MESC has an antidepressant-like action since it can significantly ameliorate the behavioral despair of depressed animals (Figures 4A, B).

### 4-Methylesculetin attenuated LPS-induced oxidative stress

**FIGURE 5:**
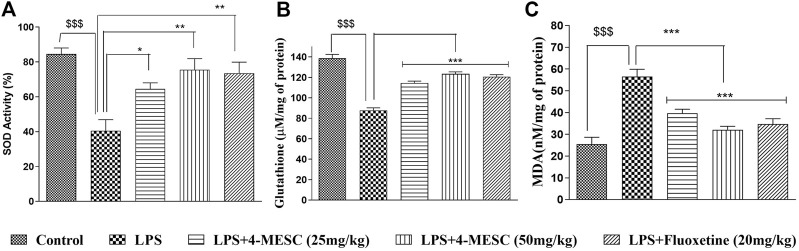
4-MESC reduces the LPS-induced oxidative stress. **(A)** Superoxide activity is evaluated with or without LPS and LPS along with 4-MESC and fluoxetine taken as a positive control. **(B)** Glutathione levels were estimated after the LPS treatment and along with 4-MESC. **(C)** Lipid peroxidation (MDA) was also checked after treatment with LPS and in the presence of 4-MESC. Data were represented as mean ± SD and *p < 0.05, ** 0.05 < *p* < 0.01, and *** 0.01 < *p* < 0.001 were considered significant compared to the LPS control.

Here, we observed that in the case of the LPS-treated group, the SOD activity and glutathione level were significantly (*p* < 0.001) decreased by 40% and 39%, respectively, as compared to the normal control. However, interestingly, the SOD activity of the 4-MESC group (25 mg/kg and 50 mg/kg) is increased significantly (*p* < 0.05 and *p* < 0.01) as compared to that of the LPS group (Figure 5A). Similarly, in the 4-MESC group (25 mg/kg and 50 mg/kg) and the standard group (fluoxetine), the glutathione level was significantly (*p* < 0.001) increased as compared to that of the LPS control group (Figure 5B). In the LPS-treated group, the MDA level significantly (*p* < 0.001) increased to 55% compared to the normal control, but significantly (*p* < 0.001) decreased to 45% in the 4-MESC-treated group (25 mg/kg and 50 mg/kg) and standard group (Fluoxetine) as compared to LPS control (Figure 5C). These studies revealed that it plays a very important role in the defense mechanism against oxidative stress (Figure 5).

### 4-Methylesculetin reduces pro-inflammatory cytokine production and enhances neuroprotection

Inflammation plays a vital role in brain-related disorders such as depression. NLRP3 inflammasome activation is also associated with the increased production of inflammatory cytokines, such as IL-6 and TNF-α. Therefore, it is necessary to determine the effect of 4-MESC on LPS-induced inflammation.

**FIGURE 6:**
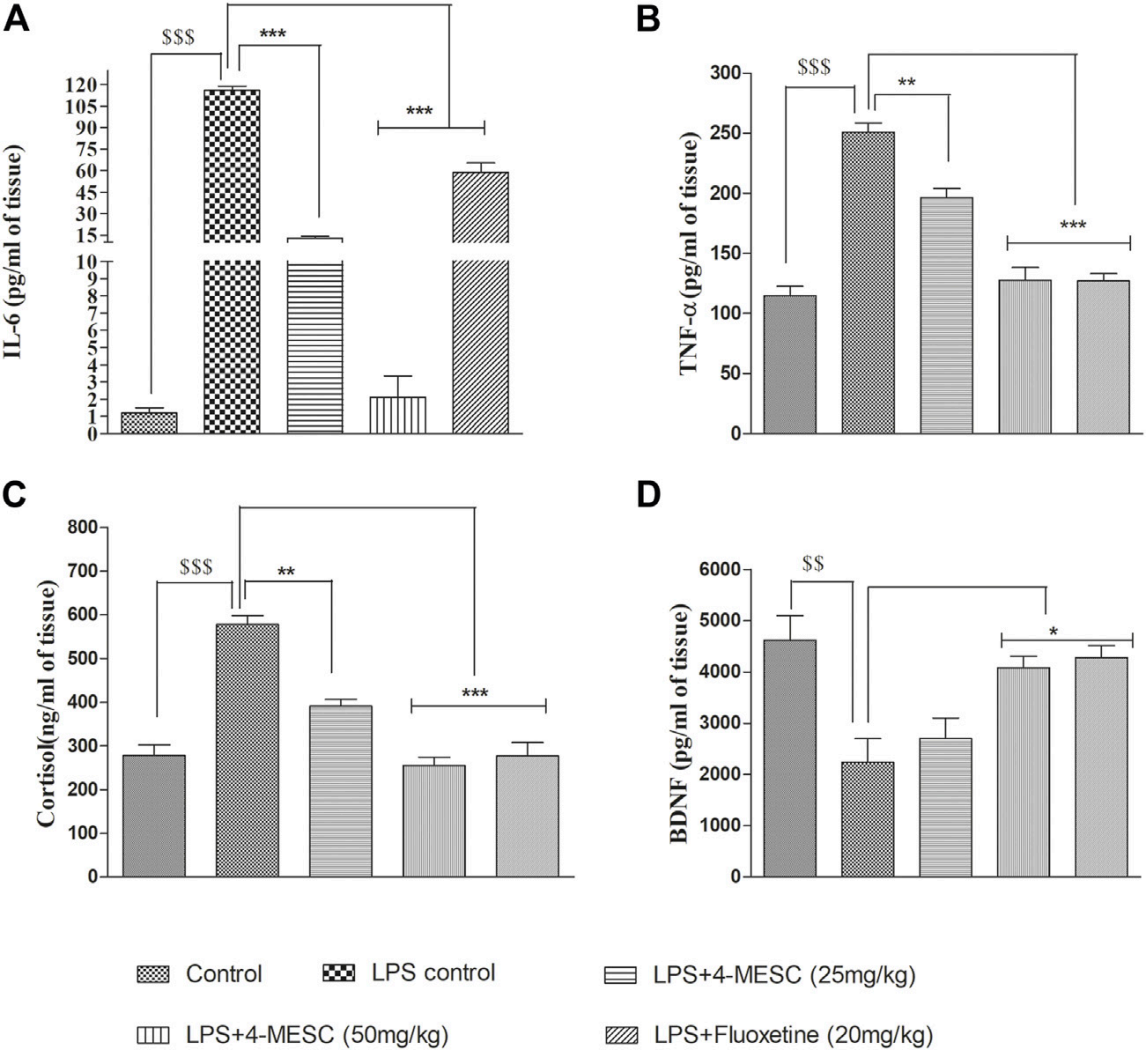
Effect of 4-MESC on pro-inflammatory cytokines. **(A)** Estimation of the IL-6 level through ELISA. **(B)** Determination of the level of TNF-α. **(C)** Neuroprotection evaluation of 4-MESC by estimation of BDNF using ELISA. **(D)** Cortisol level estimation. Data were represented as mean ± SD and *p < 0.05, ** 0.05 < *p* < 0.01, and *** 0.01 < *p* < 0.001 were considered significant.

We found that in the LPS control group, the levels of IL-6 and TNF-α significantly (*p* < 0.001) increased as compared to the normal control group, whereas in the 4-MESC group (25 mg/kg and 50 mg/kg) and standard group (fluoxetine), IL-6 decreased significantly (*p* < 0.001) as compared to the LPS control group (Figure 6A). Likewise, TNF-α also decreased significantly (*p* < 0.01 and *p* < 0.001) as compared to the LPS control at 25 mg/kg and 50 mg/kg of the 4-MESC group, respectively. Also, the efficacy was compared to the fluoxetine group, where we observed that fluoxetine reduced significantly (*p* < 0.001) with the level of TNF-α as compared to the LPS control group (Figure 6B). Furthermore, the neuroprotection effect of 4-MESC was evaluated by estimating the brain-derived neurotrophic factor. Our findings also confirmed that LPS-induced neuroinflammation significantly (*p* < 0.001) reduced the expression of BDNF as compared to the normal control group. However, the pretreatment with 4-MESC (50 mg/kg) and fluoxetine significantly (*p* < 0.05) increased the level of BDNF compared to the LPS group and exhibited its neuroprotection activity (Figure 6D). Cortisol level estimation may be the benchmark for the assessment of depression-like behavior. In the present study, LPS treatment-induced neuroinflammation was also significantly (*p* < 0.001) associated with the enhanced level of cortisol as compared to the normal control group, but significantly (*p* < 0.001 and *p* < 0.01) reduced by the 4-MESC treatment at 50 mg/kg and 25 mg/kg as compared to the LPS treatment group, respectively. A fluoxetine treatment also significantly (*p* < 0.001) decreased the level of cortisol (Figure 6C).

### 4-MESC suppressed NLRP3 inflammasome activation in gene expression studies using PCR

**FIGURE 7:**
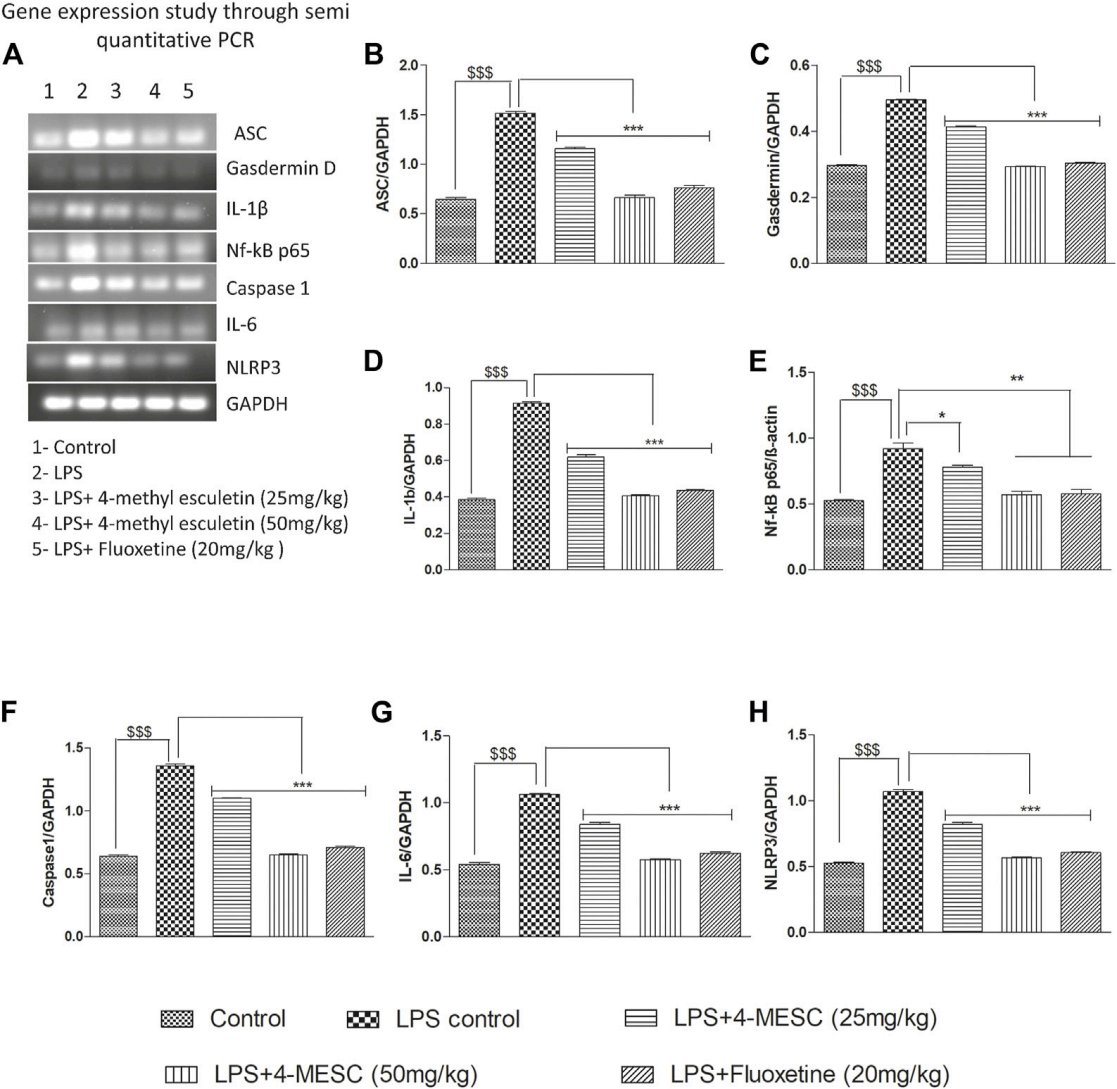
Effect of 4-MESC on gene expression involved in the NLRP3 inflammasome pathway evaluated through semi-quantitative PCR. **(A)** Representative agarose gel image of different genes, where lane 1, 2, 3, 4, and 5 represent the control, LPS, LPS +25 mg/kg of 4-MESC, LPS +50 mg/kg of MESC, and LPS+20 mg/kg of the fluoxetine group, respectively. **(B)** ASC, **(C)** caspase-1, **(D)** gasdermin D, **(E)** IL-1b, **(F)** NLRP3, **(G)** IL-6, and **(H)** Nf-κB.

To assess the 4-MESC effect on NLRP3 inflammasome activation in the hippocampus, a semi-quantitative PCR was performed to check the gene expression profile. As compared with the control group, LPS mice showed an aggravated level of nuclear NF-κBp65 (*p* < 0.001) in the hippocampus and significantly increased the expression levels of ASC (*p* < 0.001), NLRP3 (*p* < 0.001), IL-6 (*p* < 0.001), IL-1β (*p* < 0.001), and gasdermin D (*p* < 0.001) genes, which indicated the stimulation of the NLRP3 inflammasome after LPS administration having a tendency to enhance the cleavage of caspase-1. However, pretreatment with 4-methylesculetin (25 mg/kg and 50 mg/kg) and fluoxetine reversed the LPS effect and significantly alleviated the expression of NLRP3 (*p* < 0.001), ASC (*p* < 0.001), IL-1b (*p* < 0.001), IL-6 (*p* < 0.001), and gasdermin D (*p* < 0.001) (Figures 7A–H) which were upregulated by LPS. These findings demonstrated that 4-MESC was capable of alleviating the NLRP3 Inflammasome activation driven by LPS-induced pro-inflammatory cytokines release and showed an antidepressant-like effect.

### Evaluation of the NLRP3 inflammasome inhibition of 4-MESC through Western blot

**FIGURE 8:**
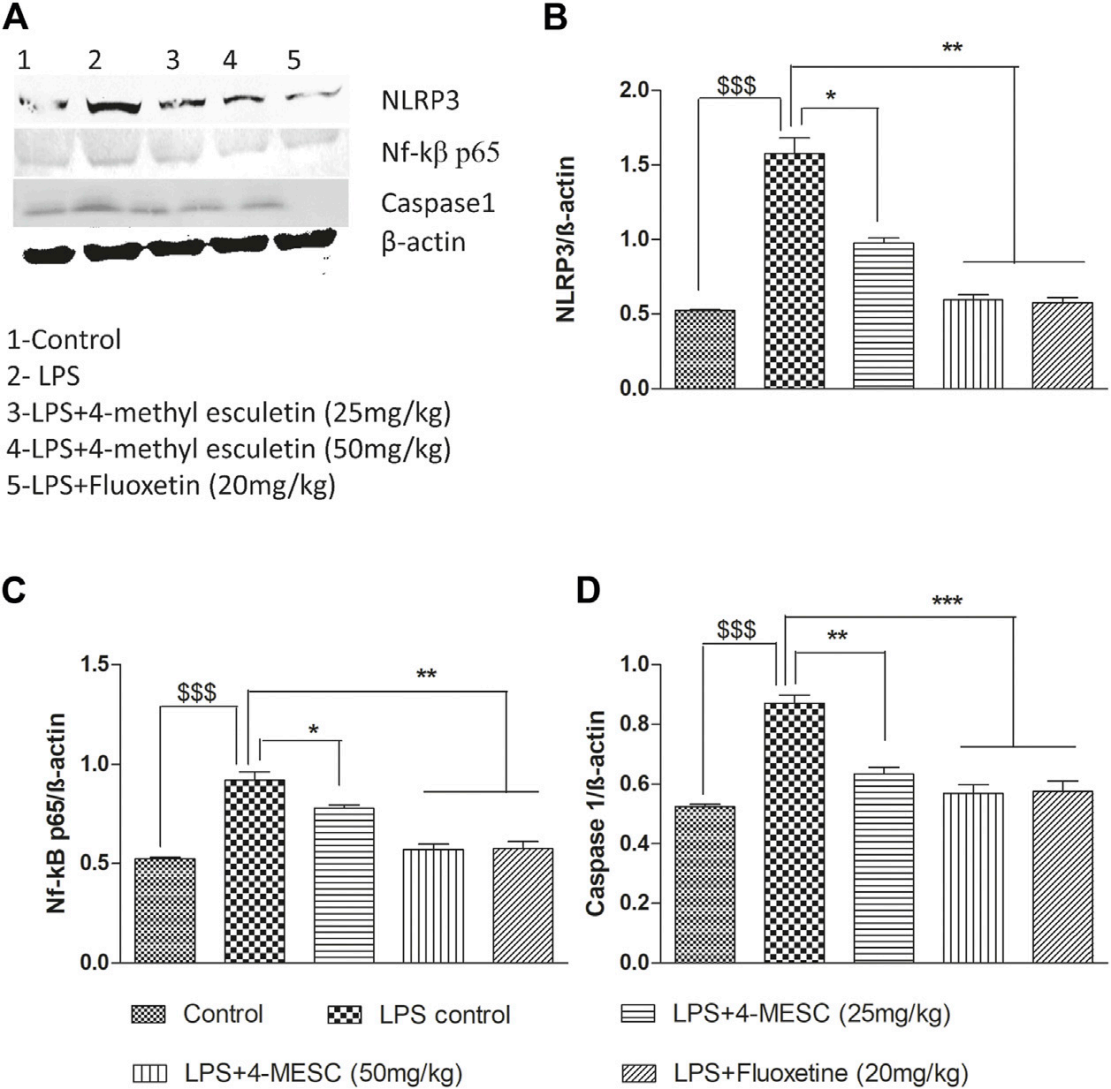
Effect of 4-MESC on expression involved in the NLRP3 inflammasome pathway evaluated using western blot. **(A)** Representative western blot image of different proteins, where lane 1, 2, 3, 4, and 5 represent the control, LPS, LPS +25 mg/kg of 4-MESC, LPS +50 mg/kg of MESC, and LPS+20 mg/kg of fluoxetin group, respectively. **(B)** NLRP3, **(C)** caspase-1, **(D)** Nf-κB. b-actin was taken as the loading control.

To further investigate the NLRP3 inflammasome suppression by 4-MESC, western blotting was used to examine the expression study of proteins such as NLRP3, Nf-kBp65, caspase 1, and b-actin. In the LPS-treated group, NLRP3 (*p* < 0.001), Nf-κBp65 (*p* < 0.001), and caspase 1 (*p* < 0.001) were expressed more than in the control group. The expression levels of NLRP3 and Nf-κBp65 are significantly (*p* < 0.05 and *p* < 0.01) reduced as compared to the LPS group with pretreatment of 4-MESC at 25 mg/kg and 50 mg/kg. Caspase 1 expression also reduced significantly (*p* < 0.01 and *p* < 0.001) as compared to the LPS group at 25 mg/kg and 50 mg/kg of 4-MESC. *β*-actin was taken as a loading control (Figure 8).

### 4-MESC attenuated the NLRP3 level

**FIGURE 9:**
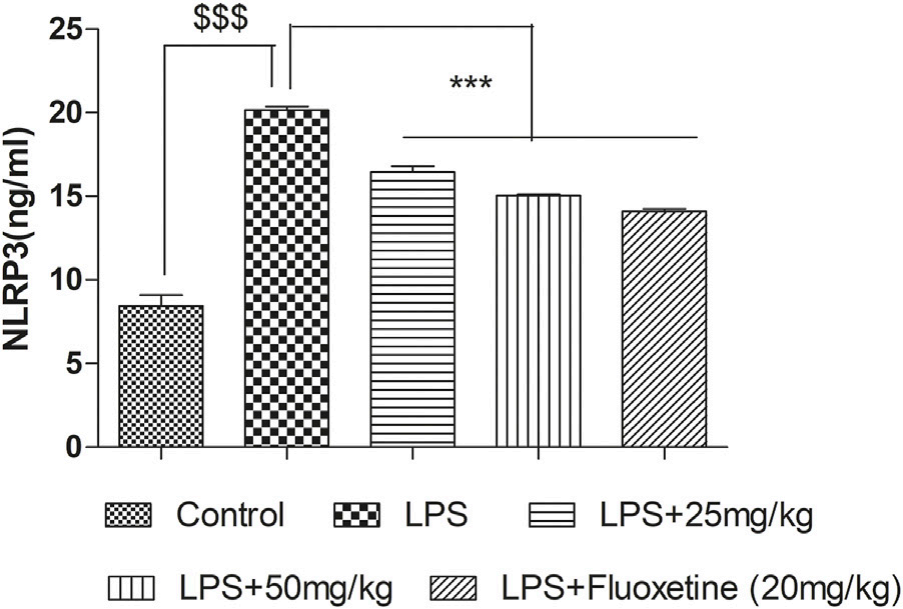
Effect of 4-MESC on NLRP3 level evaluated through ELISA Test.

In the LPS-treated group, the NLRP3 level significantly (*p* < 0.001) increased as compared to the control group. Pretreatment with 4-MESC (25 mg/kg and 50 mg/kg) and fluoxetine significantly (*p* < 0.001) reduced the NLRP3 level as compared to the LPS group (Figure 9).

## Discussion

Depression is becoming more common because of the increased stress in our daily lives in recent decades. Stress has long been recognized as a significant contributor to the pathophysiology of depression. Stress is responsible for CRF release, which subsequently activates the HPA axis and leads to depression. The deficiency of monoamines at the synaptic cleft is a major contributing factor for depression. Inflammatory responses also play an important role in the pathogenesis of depression. The LPS-induced depression model reflects a sickness-like behavior which applies to both genders. To prevent variation in results, we used only a single gender, i.e., female mice. *In silico* studies were performed to check binding affinity towards NLRP3 PYD. *In vitro* studies, such as DPPH, MTT, and ROS, were performed, and *in vivo* studies of behavioral studies such as the open-field test (OFT), Forced swim test (FST), and tail-suspension test (TST) were performed to check the anti-depressant effect of 4-MESC. Anti-oxidant properties were evaluated by the detection of the level of SOD, glutathione, and MDA through ELISA. Also, ELISA-based studies were also performed to check the level of pro-inflammatory cytokines, such as IL-6 and TNF-α, along with BDNF and cortisol. Gene expression studies were performed by the semi-quantitative and western blot techniques.

Our findings revealed that LPS induces stress, which causes depression-like behavior along with decreased expression of BDNF in the LPS group. There is proof that emotional and physical stressors can trigger inflammatory and immunological responses, which can lead to depression (Iwata et al., 2013). HPA axis dysregulation was proposed as the fundamental mechanism of IL-1’s participation in depression, and IL-1b was shown to affect the HPA axis at all levels (Pan et al., 2014). The NLRP3 inflammasome requires two signals to be activated (Pan et al., 2014). A precondition for inflammasome activation, the initial signal is produced by endogenous chemicals or microbes that cause NF-κB to be activated, increasing the production of NLRP3 and proIL-1b (Goshen and Yirmiya, 2009). The second signal causes caspase-1 to be cleaved directly by activating the NLRP3 inflammasome, which in turn causes the maturation of the pro-inflammatory cytokines IL-1b and IL-18 (Bauernfeind et al., 2009). For instance, the previous report depicted the role of the NLRP3 inflammasome in LPS-induced depression (Zhang et al., 2014). In the current investigation, we discovered that 4-MESC decreased the expression of NLRP3, ASC, caspase 1, and proIL-1b which was LPS-induced. These findings show that 4-MESC prevents the NLRP3 inflammasome from being activated. It is thought that the common activator of the NLRP3 inflammasome is the generation of ROS (Schroder and Tschopp, 2010), and also in cells, ROS production is one of the major factors for the stimulation of the apoptosis pathway (Alcocer-Gómez et al., 2016). LPS administration increases the ROS production, but pretreatment with 4-MESC significantly reduces ROS generation. Superoxide dismutase (SOD) enzyme plays a key factor in the anti-oxidant mechanism against oxidative stress in the body. This enzyme has a defensive role in treating illness caused by reactive oxygen species. The enzymes catalyzed the conversion of reactive species, such as free radicals (O_2_^−^) into stable molecular oxygen and hydrogen peroxide, and hence showed an anti-oxidant effect (T. Wang et al., 2004). In a similar way, GSH is essential for maintaining redox equilibrium, shielding cells against oxidative damage and the toxicity of xenobiotic electrophiles (Forman et al., 2009). In the case of an inflammatory condition, the SOD activity and glutathione level are compromised and the MDA level is increased. Therefore, it is necessary to check the effect of compounds on SOD activity and levels of glutathione and MDA. Here, we found that the 4-MESC significantly enhances the SOD activity and increase the level of glutathione and decrease the level of MDA as compared to the LPS-treated group which reveal its anti-oxidant properties. Additionally, we demonstrated that 4-MESC inhibits central NLRP3 inflammasome activation, which ameliorates the LPS-induced depressive behavior. Numerous studies show that the NLRP3 inflammasome is essential for the development of depression (Martinon, 2010). The NLRP3 inflammasome may therefore constitute a new therapeutic target for the treatment of depression. In this study, we used a mouse model of depression generated by LPS and discovered that LPS activated the NLRP3 inflammasome in the hippocampal region. The systemic administration of LPS resulted in illness and moderate depressive-like behavior, according to our behavioral data. In the current rodent model, it was not able to distinguish between illness and depressive-like behavior due to the overlapping time courses and moderate effects on behavior that is individually associated with depression (Biesmans et al., 2013). With less depressive behavior, including a significant reduction in immobility duration in the TST and FST without affecting spontaneous locomotor activity in OFT, 4-MESC therapy eliminated LPS-induced activation of the NLRP3 inflammasome. These findings imply that the depressed behaviors seen in 4-MESC-treated mice may be attributed to the inhibition of cerebral NLRP3 inflammasome activation. Also, *in silico* studies (docking and MD simulation) showed that 4-MESC strongly binds to the NLRP3 PYD, further proving its NLRP3 inflammasome inhibition. Inflammation has been linked to the etiology of depression, according to the accumulated data, and people with depression have consistently been seen to produce more inflammatory cytokines (Slavich & Irwin, 2014). We examined levels of few inflammatory cytokines in the serum and inflammation-related proteins in the hippocampus in order to investigate the potential mechanism of action of antidepressant 4-MESC. We discovered that LPS administration increased the serum levels of inflammatory cytokines such as TNF-α and IL-6, which have been linked to the etiology of depression. Our findings demonstrated that 4-MESC could successfully decrease the levels of TNF-α and IL-6 caused by LPS, indicating its effectiveness against neuroinflammation. BDNF aids and supports the survival of existing neurons and also stimulates the maturation and differentiation of new neurons (Acheson et al., 1995). Inflammation regulates the BDNF expression in the brain (Acheson et al., 1995). Our findings suggest that LPS administration decreases the expression of BDNF in the brain tissue. However, pretreatment with 4-MESC significantly enhanced the level of BDNF, which showed its neuroprotective nature. Cortisol levels are elevated in the depression-like behavior (Acheson et al., 1995). The LPS treatment caused neuroinflammation and increased cortisol levels in the LPS-treated group but decreased them in the 4-MESC-treated group. The compound 4-methylesculetin is shown to be safe in previous studies on acute toxicity studies with an LD50 value of 3,200 mg/kg, *p.o*. on mice (Hemshekhar et al., 2013). The observed beneficial effect in this model complements its potential to be a safer antidepressant agent.

## Conclusion

In the present study, we evaluated the antidepressant effect of 4-MESC through the inhibition of LPS-induced NLRP3 inflammasome activation. The *in vitro* study showed the safety of the compound and prevention of ROS, along with DPPH inhibition. All of these findings indicated that this molecule has the potential to be useful in inflammasome inhibition. The *in vivo* study reflected the improvement in immobility time induced by LPS. This behavioral parameter was supported by various molecular markers where the compound showed an increase in BDNF expression, which was decreased due to LPS. Our results showed that 4-MESC effectively reduced the levels of TNF-α, IL-6, and cortisol induced by LPS, demonstrating the compound’s effect on neuroinflammation. Overall, our study demonstrated the antidepressant effect of 4-MESC through the inhibition of NLRP3 inflammasome activation, suggesting the potential clinical use of 4-MESC in NLRP3 inflammasome-associated inflammatory diseases such as depression.

## Data Availability

The original contributions presented in the study are included in the article/supplementary materials, further inquiries can be directed to the corresponding author.
